# Supplementary material for: Regulation of NTRK2 alternative splicing by PRPF40B controls neural differentiation and synaptic plasticity
Source: Cell Death Dis. 2025 Dec 8;17(1):73. doi: 10.1038/s41419-025-08301-9 (PMC12827282; doi:10.1038/s41419-025-08301-9)

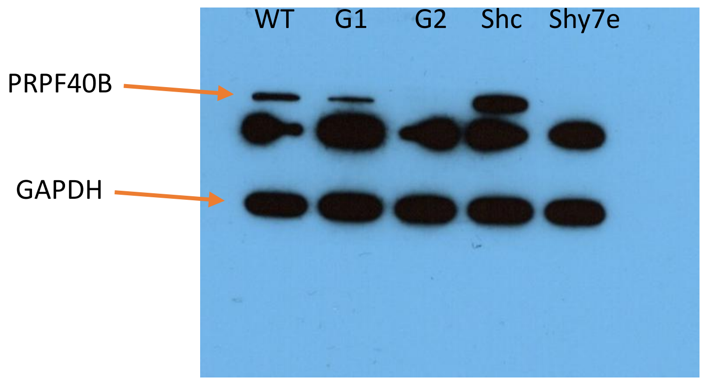

FIGURE 1-A AND FIGURE S1-A

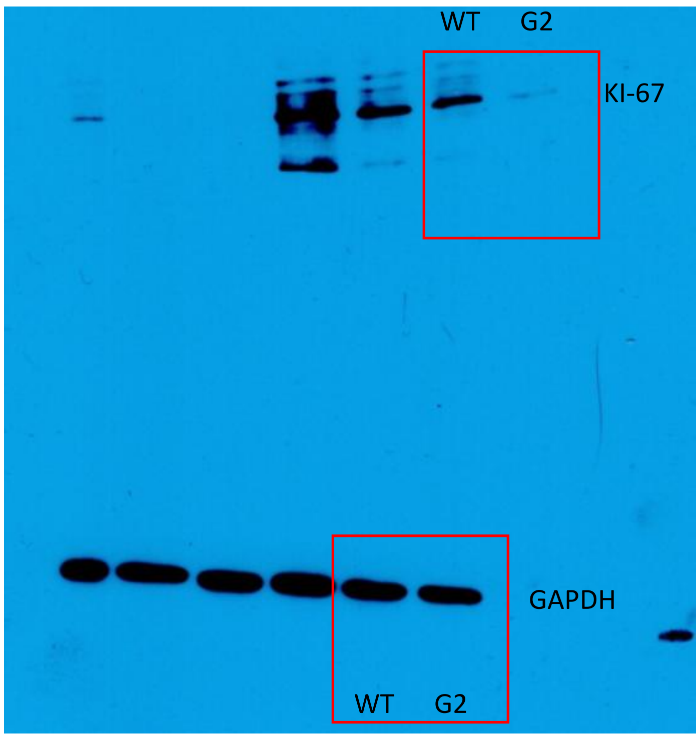

FIGURE 1-B

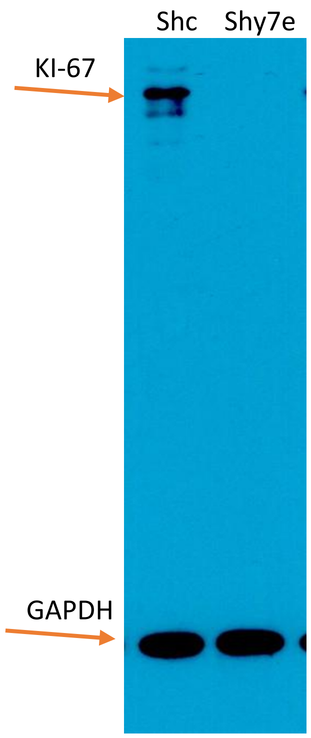

FIGURE S1-B

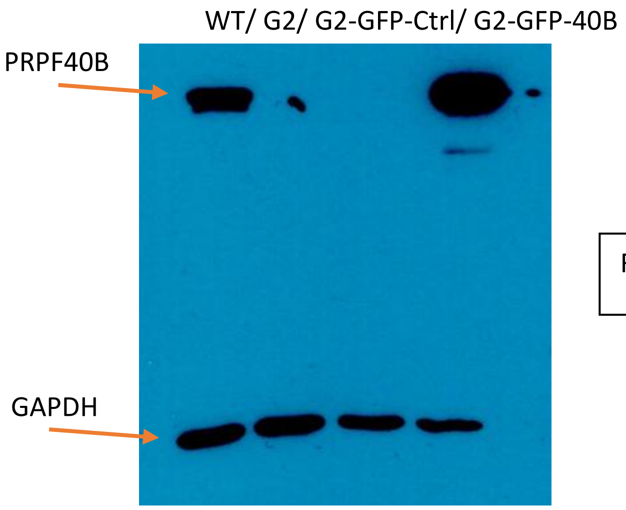

FIGURE S2-B

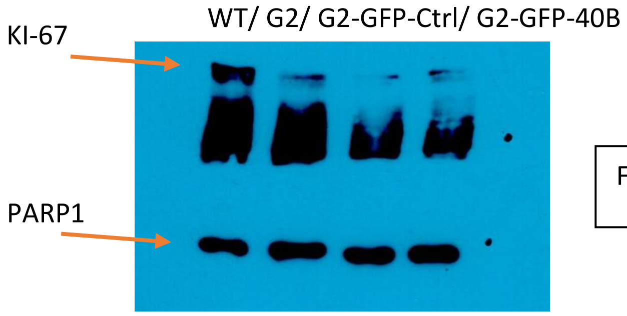

FIGURE S2-D

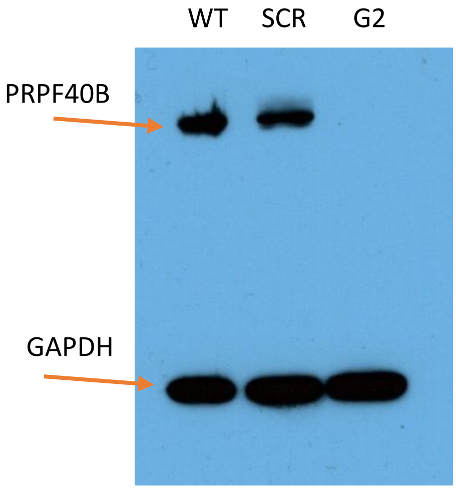

FIGURE 3-A

FIGURE 5-B

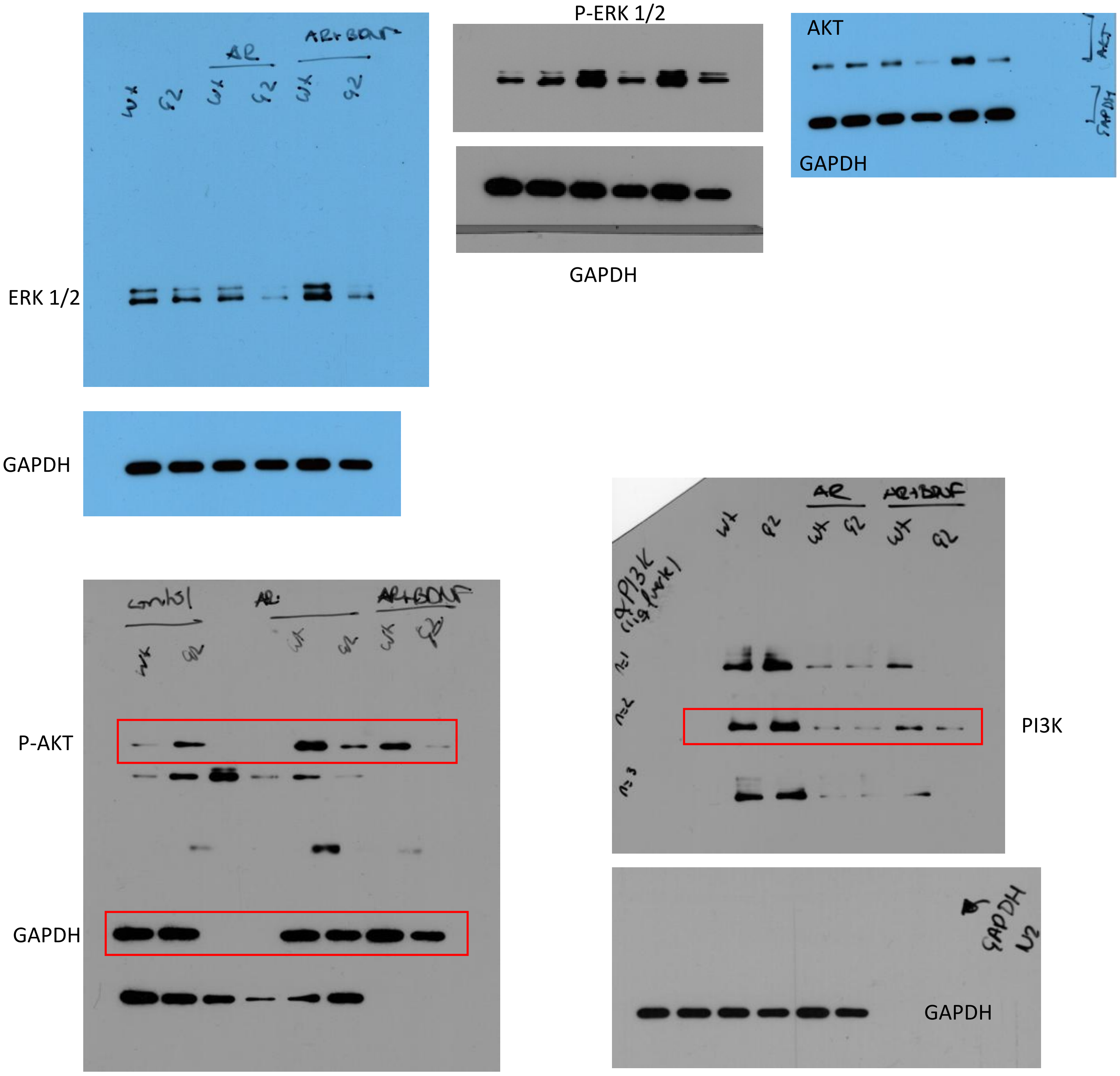

FIGURE 5-C

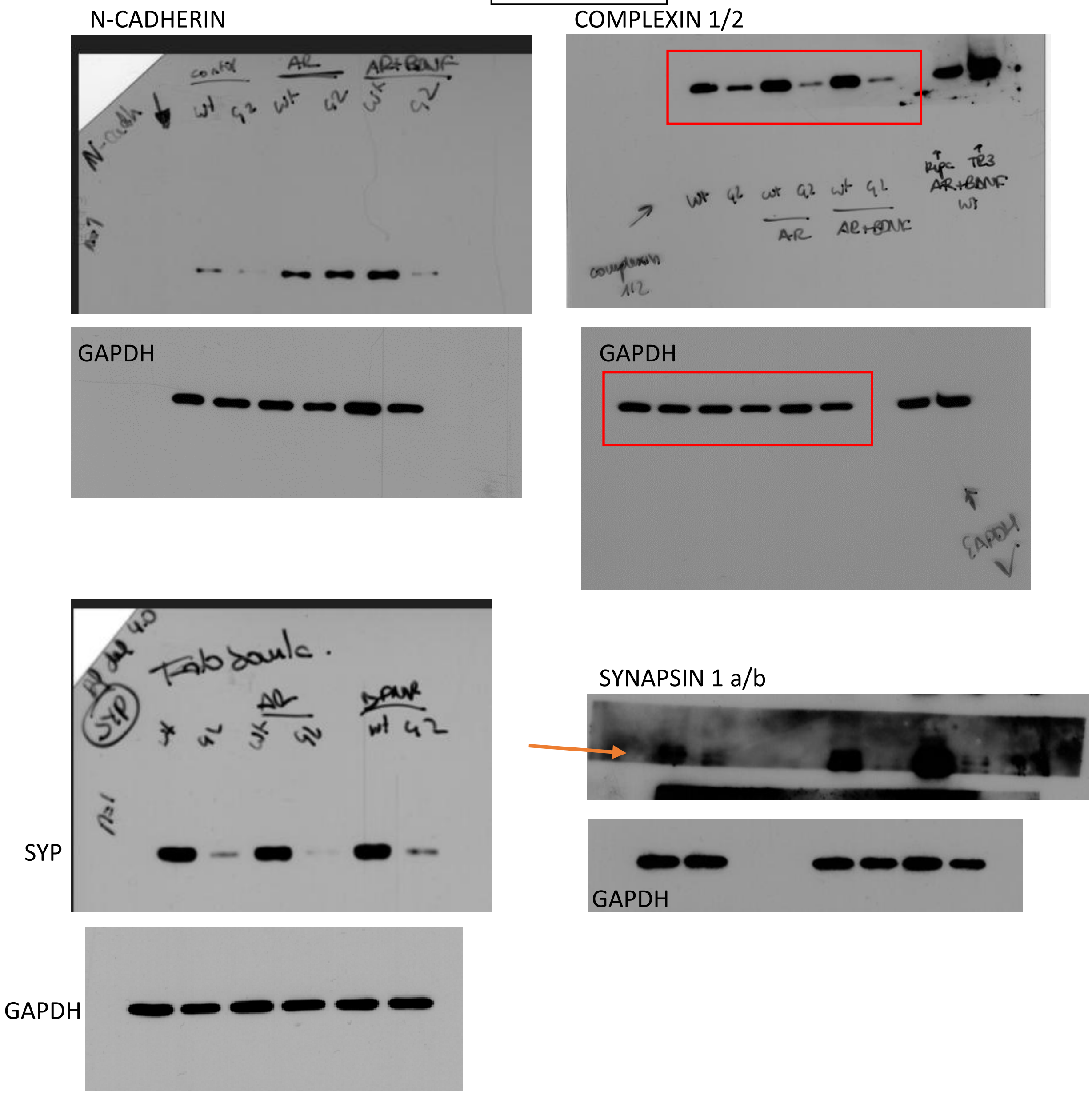
N-CADHERIN
COMPLEXIN 1/2
GAPDH
GAPDH
SYNAPSIN 1 a/b
SYP
GAPDH
GAPDH

FIGURE 5-D

GAPDH
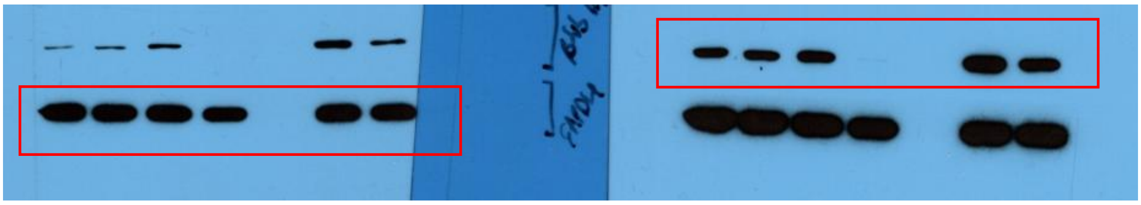
B-TUBULIN III

DREBRIN
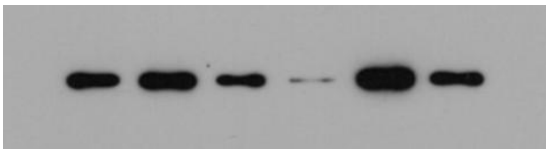

GAPDH
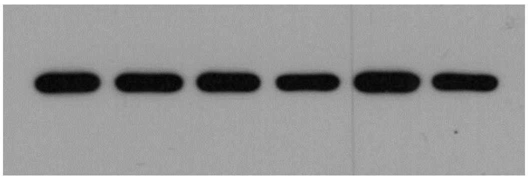

FIGURE 5-E

PRPF40B
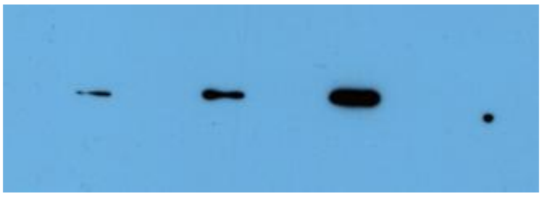

GAPDH
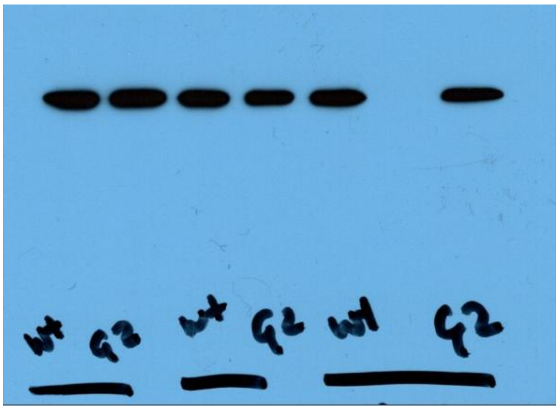

FIGURE 6-B

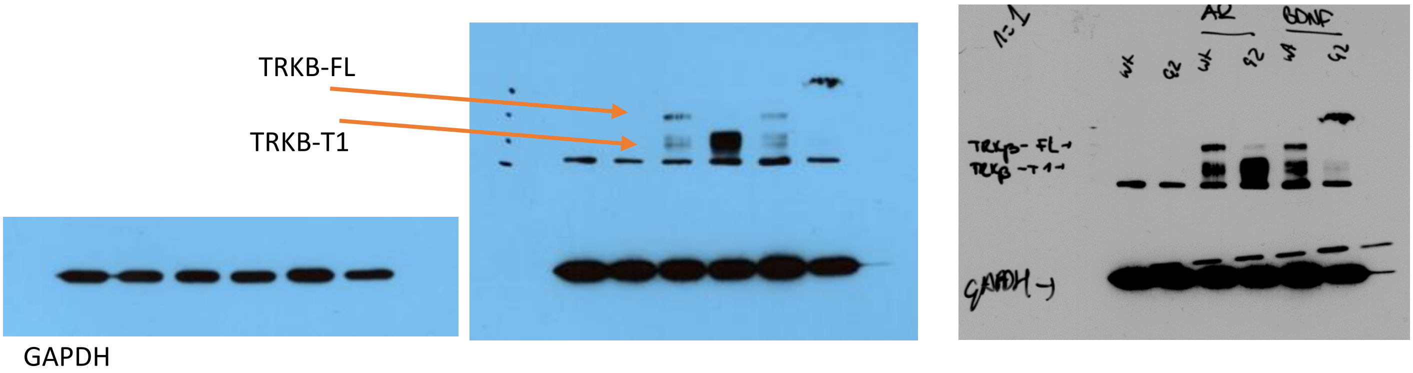

FIGURE 7-B

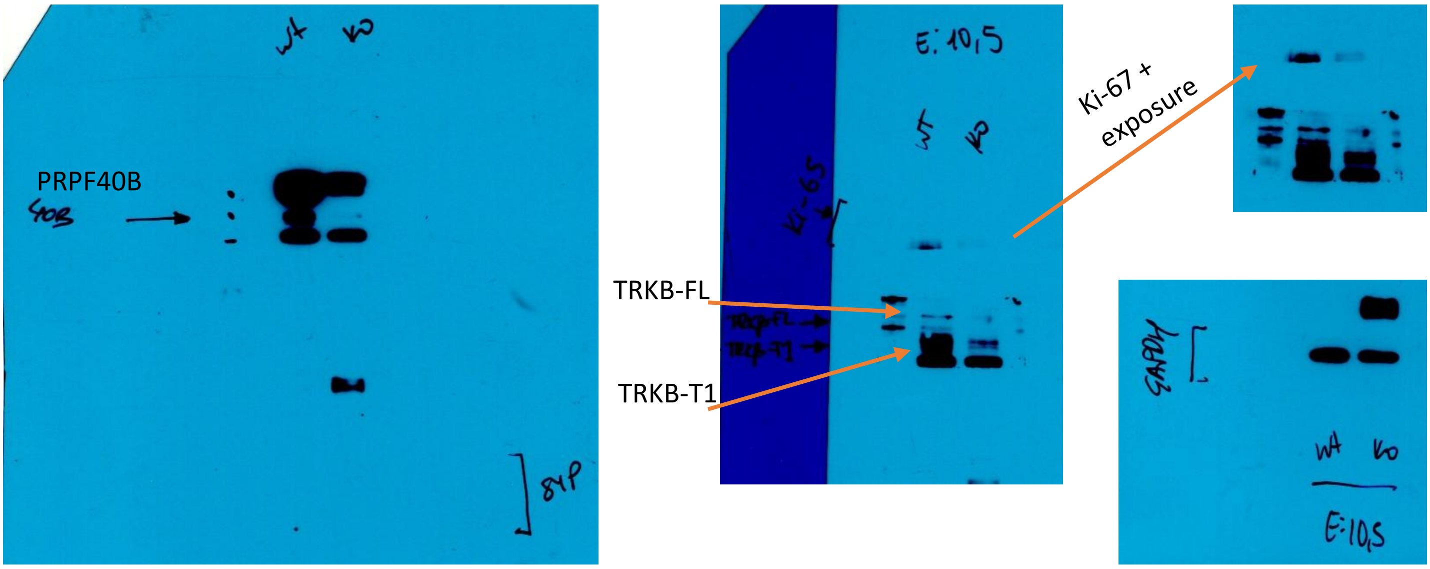

FIGURE 7-C

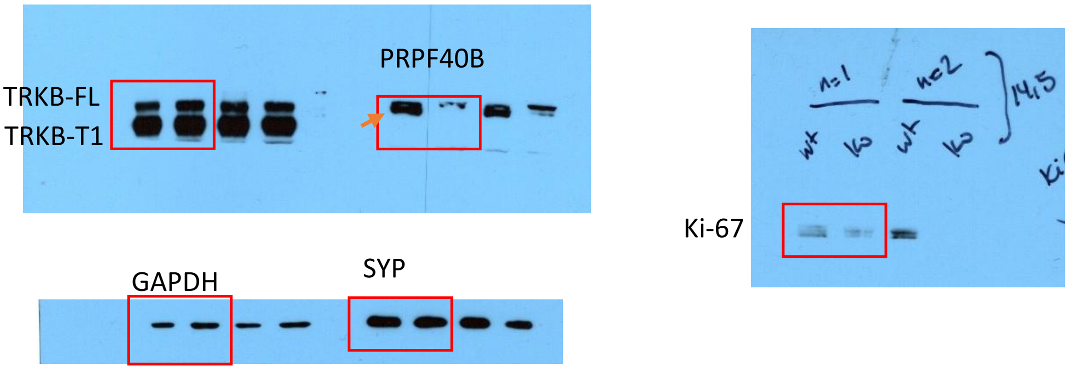

FIGURE S8-B

HIGH EXPOSURE

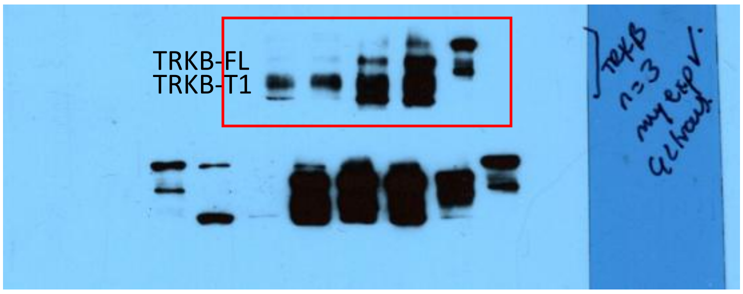

LOW EXPOSURE

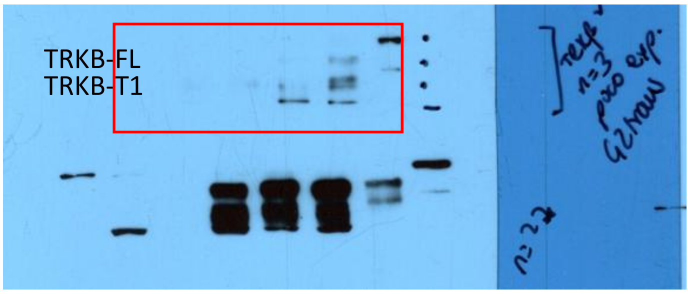

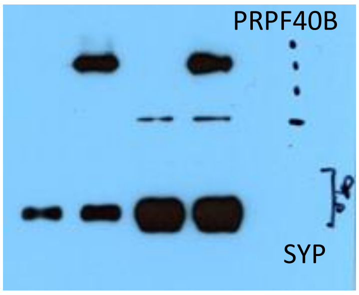

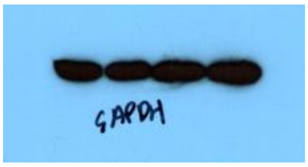

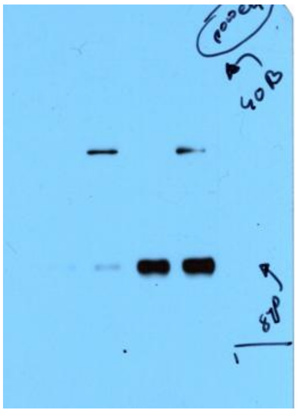

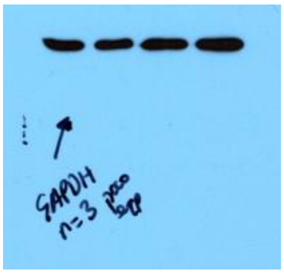

FIGURE S8-C

HIGH EXPOSURE

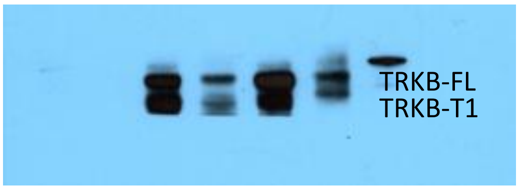

LOW EXPOSURE

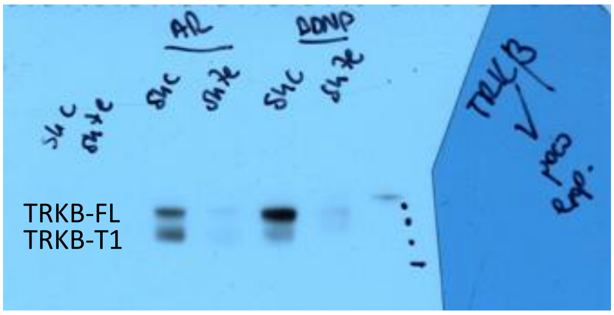

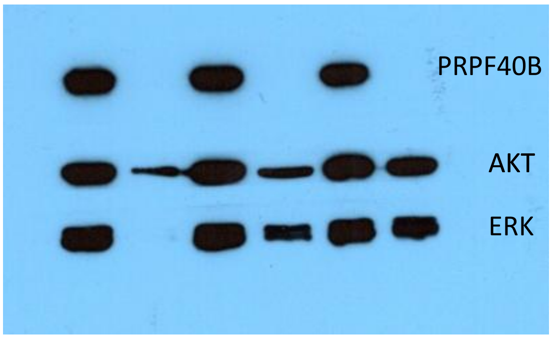

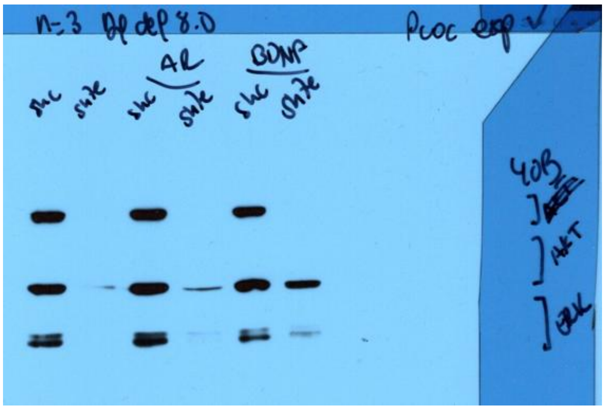

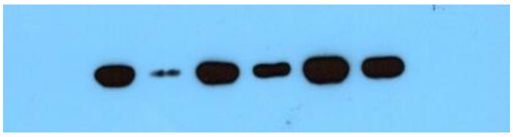

SYP

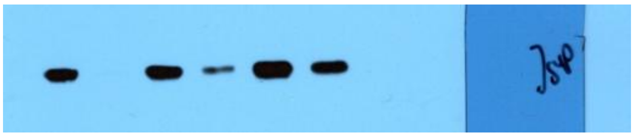

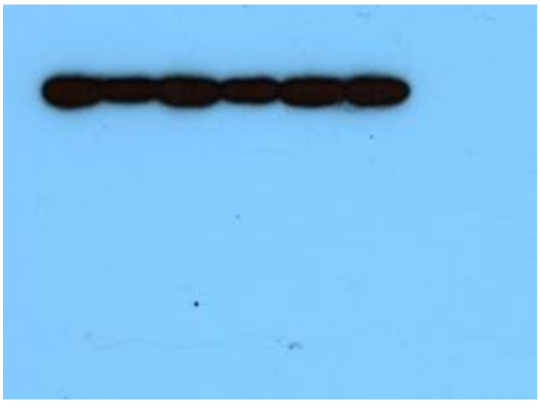

GAPDH

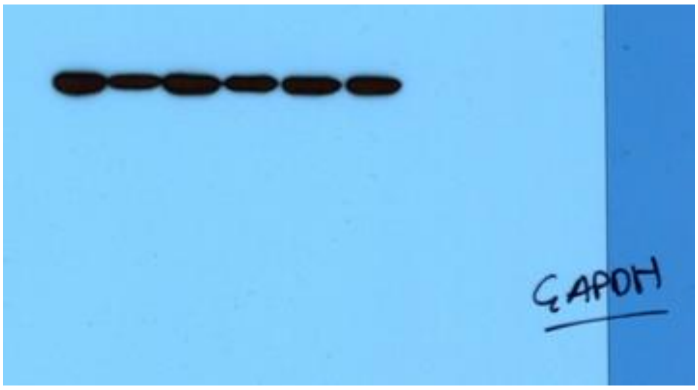

Supplement: Supplementary file 1 — Supplemental Material (uncroppedWB) [file 41419_2025_8301_MOESM1_ESM.pdf]
